# Sabizabulin, a Potent Orally Bioavailable Colchicine Binding Site Agent, Suppresses HER2+ Breast Cancer and Metastasis

**DOI:** 10.3390/cancers14215336

**Published:** 2022-10-29

**Authors:** Raisa I. Krutilina, Kelli L. Hartman, Damilola Oluwalana, Hilaire C. Playa, Deanna N. Parke, Hao Chen, Duane D. Miller, Wei Li, Tiffany N. Seagroves

**Affiliations:** 1 Department of Pathology, College of Medicine, University of Tennessee Health Science Center, Memphis, TN 38103, USA; 2 Department of Pharmaceutical Sciences, College of Pharmacy, University of Tennessee Health Science Center, Memphis, TN 38103, USA; 3 Center for Cancer Research, University of Tennessee Health Science Center, Memphis, TN 38103, USA; 4 Drug Discovery Center, College of Pharmacy, University of Tennessee, Memphis, TN 38103, USA

**Keywords:** HER2^+^ breast cancer, colchicine binding site inhibitor, orally available tubulin inhibitor, patient-derived xenograft, tumor metastasis

## Abstract

**Simple Summary:**

The sabizabulin agent represses tumor cell growth, cell migration, and colony formation, and induces cell death in HER2+ breast cancer models. Sabizabulin is comparable to paclitaxel to suppress HER2+ xenograft growth and to inhibit lung metastasis in a HER2+ patient-derived xenograft (PDX) model. Sabizabulin is a promising orally available agent as an alternative to taxanes to target tubulin in breast cancer patients, including the HER2+ breast cancer molecular subtype.

**Abstract:**

HER2+ breast cancer accounts for 15% of all breast cancer cases. Current frontline therapy for HER2+ metastatic breast cancer relies on targeted antibodies, trastuzumab and pertuzumab, combined with microtubule inhibitors in the taxane class (paclitaxel or docetaxel). It is well known that the clinical efficacy of taxanes is limited by the development of chemoresistance and hematological and neurotoxicities. The colchicine-binding site inhibitors (CBSIs) are a class of promising alternative agents to taxane therapy. Sabizabulin (formerly known as VERU-111) is a potent CBSI that overcomes P-gp-mediated taxane resistance, is orally bioavailable, and inhibits tumor growth and distant metastasis in triple negative breast cancer (TNBC). Herein, we demonstrate the efficacy of sabizabulin in HER2^+^ breast cancer. In vitro, sabizabulin inhibits the proliferation of HER2+ breast cancer cell lines with low nanomolar IC_50_ values, inhibits clonogenicity, and induces apoptosis in a concentration-dependent manner. In vivo, sabizabulin inhibits breast tumor growth in the BT474 (ER+/PR+/HER2+) xenograft model and a HER2+ (ER-/PR-) metastatic patient-derived xenograft (PDX) model, HCI-12. We demonstrate that sabizabulin is a promising alternative agent to target tubulin in HER2+ breast cancer with similar anti-metastatic efficacy to paclitaxel, but with the advantage of oral bioavailability and lower toxicity than taxanes.

## 1. Introduction

Worldwide, there are 2.26 million new cases of breast cancer, resulting in 685,000 deaths per year [1]. Death is primarily due to the expansion of metastatic lesions in response to chemoresistance. Breast cancer is classified into four molecular subtypes based on estrogen receptor (ER), progesterone receptor (PR), and human epidermal growth factor 2 (HER2/ERBB2) receptor status: luminal A (ER+/PR+/HER2−), luminal B (ER+/PR+/HER2+), HER2-overexpressing, and triple negative breast cancer (TNBC; ER^-^/PR-/HER2−) [2,3,4]. Common treatment strategies include surgical resection of the primary tumor, combined with neo-adjuvant/adjuvant chemotherapy, and radiotherapy. Up to 30% of women diagnosed with breast cancer will relapse within 30 years [5,6], in part through acquired resistance to chemotherapy agents.

HER2+ breast cancers account for up to 15% of all cases (~10% ER+/PR+/HER2+; ~5% ER−/PR−/HER2+) [7,8]. HER2 signaling is triggered by dimerization of receptors, activating cell survival, proliferation, and invasive processes through the RAS pathway via PI3K/Akt or MAPK [7]. Current therapies for HER2+ patients rely on antibody therapy and antibody-drug conjugates (T-DM1) to inhibit receptor dimerization and tumor progression or small molecules to block receptor tyrosine kinase signaling. Patients with HER2+ breast cancer, especially the non-luminal cases, have a poorer prognosis, similar to TNBC, with higher histological tumor grades, higher rates of recurrence, and more frequent distant metastases to bone, liver, lung, and brain than patients with luminal breast cancer [9,10,11,12,13].

Frontline therapy for HER2+ breast cancer relies on extracellularly targeted anti-HER2 antibodies (Herceptin/trastuzumab and Perjeta/pertuzumab) that inhibit downstream HER2-dependent tyrosine kinase signaling pathways, facilitating antibody-dependent cell-mediated cytotoxicity [14,15,16,17,18]. A comprehensive body of work has shown anti-HER2 targeting agents are most effective in combination with cytotoxic systemic chemotherapy drugs, including taxanes [19,20,21,22,23]. Data from the landmark phase-III CLEOPATRA study further demonstrated that trastuzumab or pertuzumab, when paired with a taxane, significantly improved progression-free survival and overall survival, establishing this regimen as a preferred therapy for HER2+ patients [24,25,26,27]. These studies led to the current recommendation for frontline therapy of pairing a taxane with traszutumab and pertuzumab antibodies for HER2+ metastatic disease [28]. Lapatinib, an oral tyrosine kinase inhibitor, is currently part of a second-line treatment plan for metastatic HER2+ breast cancer patients who have already had treatment with an antibody- and chemotherapy-based regimen. Lapatinib significantly improved clinical outcomes when paired with paclitaxel [27,29,30,31]. Lapatinib is typically used alongside trastuzumab or capecitabine [28]. Overall, these studies indicate that taxanes complement HER2-targeted therapy and improve disease outcomes.

Taxanes are one class of microtubule inhibitors that interfere with microtubule dynamics by binding to the taxol binding site of the tubulin subunits. There are three primary binding sites of tubulin targeting agents, the taxol-, colchicine-, or the vinca-binding sites. Targeting microtubules with agents that bind to any of these sites induces mitotic arrest and apoptosis [32]. Microtubule stabilizing agents, like taxanes (paclitaxel/Taxol and docetaxel/Taxotere), bind to the taxol-binding sites in β-tubulin and stabilize polymerized microtubules. In contrast, the majority of microtubule destabilizing agents bind to the colchicine- or vinca-binding sites to inhibit tubulin polymerization [33]. Taxanes are broadly used as standard of care agents but have several clinical limitations. First, they are good substrates of drug efflux pumps such as MDR1/P-gp/ABCB1 [33,34,35], leading to chemoresistance. Taxanes also induce dose-limiting neurotoxicity and hematopoietic toxicity [36,37]. Finally, taxanes have poor oral bioavailability, requiring the use of surfactants and intravenous infusions, which can cause side effects in patients [38].

We previously characterized the anti-cancer efficacy of sabizabulin (also known as VERU-111), a novel colchicine binding site inhibitor (CBSI), in TNBC models, including in a patient-derived xenograft (PDX) model of metastatic breast cancer (MBC) [39]. Colchicine toxicity limits its use in oncology [40]. Sabizabulin causes cell cycle arrest and apoptosis and disrupts angiogenesis to inhibit the growth of primary tumors and distant metastases [39,40,41]. Moreover, it overcomes taxane-resistance in vitro and in vivo in several cancer types, including TNBC [39,40,42,43,44]. Sabizabulin has good aqueous solubility without surfactants, shows excellent pharmacokinetic properties, is orally bioavailable, and, unlike paclitaxel, does not induce peripheral neuropathy at efficacious doses [42,45]. Moreover, recent phase 1b/II studies using sabizabulin in men with metastatic castration-resistant prostate cancer confirmed pre-clinical observations that sabizabulin does not have the typical toxicities associated with the use of taxanes (neutropenia and neuropathy), even at doses exceeding therapeutic dose levels [46]. Since the addition of taxanes to HER2-targeting therapies enhances survival, it is critical to develop and to optimize drugs for HER2+ patients that can safely circumvent taxane chemoresistance. 

In this study, we have demonstrated the in vivo efficacy of sabizabulin using two conventional HER2^+^ cell lines, BT474 (ER+/PR+/HER2+) and SKBR3 (ER−/PR−/HER2+), BT474 xenografts and a metastatic PDX HER2+ model, HCI-12 (ER−/PR−/HER2+). Sabizabulin showed strong anti-proliferative activity, inhibited clonogenicity, and induced apoptosis of tumor cells, and significantly inhibited primary tumor progression and lung metastasis. We also paired paclitaxel or sabizabulin with lapatinib in AU565 (ER−/PR−/HER2+; lapatinib-sensitive) and JIMT (ER−/PR−/HER2+; lapatinib-resistant) cells [47,48], and found that sabizabulin, but not paclitaxel, was synergistic with lapatinib to inhibit HER2+ tumor cell growth. Taken together, we conclude that sabizabulin effectively targets HER2+ metastatic breast cancer and is a promising clinical drug candidate for HER2+ patients.

## 2. Materials and Methods

### 2.1. Chemicals, Cell Culture, and PDX Materials

Colchicine was purchased from Sigma-Aldrich (C9754, purity > 95.0%, St. Louis, MO, USA), paclitaxel was purchased from LC Laboratories (P-9600, purity 99.5%, Woburn, MA, USA), lapatinib was purchased from MedKoo Biosciences (100946, purity > 99%, Morristown, NC, USA), and sabizabulin (purity > 98.0%) was synthesized according to [49]. BT474, SKBR3 and AU565 cells were obtained from the American Type Culture Collection (ATCC). Lapatinib-resistant JIMT-1BR-GFP/Luc cells (JMIT) were generously provided by Dr. Patricia Steeg at the National Cancer Institute [47]. Cells were grown in RPMI (BT474 and AU565) or DMEM-Hi (SKBR3 and JIMT) medium supplemented with 10% FBS, 1% antibiotic-antimycotic solution and 15 mM HEPES; AU565 cells were also supplemented with 2.5 μg/mL insulin. Cells were authenticated at the University of Arizona Genetics Core and routinely screened for mycoplasma using the MycoAlert kit (Lonza, Basel, Switzerland). HCI-12 PDX tumor fragments were provided by Dr. Alana Welm through the Huntsman Cancer Institute (HCI) pre-clinical research resource [50] and PDX tumor tissue was authenticated back to the original patient material. PDX tumor fragments were serially re-transplanted in the inguinal mammary fat pads of female Nod-Scid-Gamma (NSG) mice (Jackson Laboratory, Bar Harbor, ME, USA, stock #005557). 

### 2.2. Cell Proliferation, IC_50,_ and Cytotoxicity Assays in BT474 and SKBR3 Cells

Cells were seeded overnight into 96-well plates (5000 cells/well; n = 8 wells), treated the next day with increasing concentrations of sabizabulin, colchicine, or paclitaxel, and imaged with the IncuCyte S3 live-cell imager (Sartorius, Göttingen, Germany). Where indicated, Cytotox Green reagent (4633, Sartorius) was added. Phase masking algorithms were applied to determine cell confluence. Cytotoxicity was calculated as a percentage of green units overlapping with cells after applying a masking algorithm to enumerate cells. At the study endpoint, growth inhibition was measured by the MTS assay; drug response was first normalized to untreated vehicle controls and/or to initial seeding density, plotted on a log scale, and then plotted using non-linear regression best fit analysis in GraphPad Prism 9.0. The mean IC_50_ ± SEM was derived from at least 3 biological replicates.

### 2.3. Colony Formation Assay

Cells were plated into 96-well plates (500 cells/well; n = 8 replicates) and treated 24 h later with increasing concentrations of drug. Spent media was replaced every 4 days, and images were obtained every 12 h using the IncuCyte S3. The phase masking algorithm was applied to enumerate colony formation. Data were exported to Prism 9.0 and normalized to seeding density. Cells were washed with PBS, fixed with methanol, stained with 0.5% crystal violet, and imaged with EVOS FL Imaging System (Life Technologies, Carlsbad, CA, USA).

### 2.4. Protein Extraction and Western Blotting 

Cells grown to 80% confluence were incubated with increasing concentrations of sabizabulin (10, 20, 50, 100 nM) or 100 nM colchicine or paclitaxel for 24 h. For time-dependent studies, cells were exposed to 100 nM of sabizabulin for 12, 24, 48, or 72 h. Flash-frozen tumors were ground under liquid nitrogen using a mortar and pestle and homogenized. Proteins were extracted in RIPA lysis buffer as in [39] and quantitated by a Bradford assay.

To detect HER2, proteins (15 μg) were resolved on a 10% Bis-Tris gel. For cleaved PARP, phosphorylated BCL2, and cleaved caspase-3, proteins were resolved on 4–15% gradient gel (BT474, up to 40 μg) or 10% Bis-Tris BOLT gels (SKBR3, 120 μg; NW00100, Thermo-Fisher, Waltham, MA, USA). Proteins were transferred onto PVDF low-fluorescence membrane (Millipore, Burlington, MA, USA) using the eBlot L1 transfer system (L00686, GeneScript, Piscataway, NJ, USA). Membranes were blocked with 5% non-fat dry milk/TBST and probed with primary antibodies at 4 °C overnight. After extensive washing, membranes were incubated with anti-rabbit, or anti-mouse whole IgG secondary HRP-conjugated antibodies (Jackson Immunologicals, West Grove, PA, USA) at RT for 1 h and detected by ECL reagent (WBKLS0500, Millipore), followed by exposure to film.

### 2.5. Orthotopic BT474 Xenografts

All animal studies adhered to NIH Principles of Laboratory Animal care and protocols approved by the local Institutional Animal Care and Use Committee (protocol 20-0181, Seagroves). Female NSG mice (5–6 weeks old) were provided with a subcutaneous estradiol beeswax pellet (E8875, Sigma-Aldrich) 1 week prior to inoculation with BT474 cells. Cells (2.5 × 10^6^) were suspended in 20 µL HBSS:10 µL growth factor-reduced Matrigel (354230, Corning, Corning, NY, USA) and bilaterally injected into each inguinal mammary fat pad using a Hamilton syringe mounted with a 1/2″ 26G PT2 needle. Tumors were measured with digital calipers twice a week. When at least one tumor grew to 100 mm^3^, mice were randomized such that mean tumor volumes were equivalent across treatment cohorts [**1**: vehicle (diluent of paclitaxel: ethanol:Cremophor-EL:0.9%saline 1:1:18 ratio; 3×/week, IP, n = 11 mice); **2**: 10 mg/kg paclitaxel (3×/week, IP, n = 11 mice,); **3**: 17 mg/kg sabizabulin (diluent: PEG300:water = 3:7 ratio; 3×/week, PO, n = 12 mice)]. Body weights were recorded twice a week. Tumor volume was calculated as (width^2^ × length)/2. Excised tumors were photographed, and the tumor volume was calculated ex vivo by caliper measurement. Harvested tumors were bisected; viable tumor tissue was divided and flash frozen in liquid nitrogen or fixed overnight in 10% neutral-buffered formalin (NBF) and embedded in paraffin. 

### 2.6. HCI-12 PDX Model

PDX tumor fragments (~2 mm^3^) harvested from a tumor-bearing mouse were bilaterally implanted into each inguinal mammary fat pad of recipient NSG females (6–7 weeks old). Treatment cohorts were: **1**: vehicle (diluent of paclitaxel: ethanol:Cremophor-EL:0.9%saline = 1:1:18 diluent; 3×/week, IP, n = 11 mice); **2**: 10 mg/kg paclitaxel (in ethanol:Cremophor-EL:0.9%saline = 1:1:18 diluent; 3×/week, IP n = 11 mice), **3**: 20 mg/kg sabizabulin (PEG300:water = 3:7 ratio diluent; 3×/week, PO, n = 12 mice). Mice were randomized as previously described before assignment to a treatment arm. All animals were euthanized and processed as in 2.5, except that lungs were also harvested after inflation with saline and fixed for histopathology. 

### 2.7. Histology and Immunohistochemistry (IHC) Analysis

Paraffin-embedded specimens were sectioned (5–7 µm) onto positively charged slides at the UTHSC Research Histology Core. For tumor slides, antigens were retrieved in 1× citrate buffer (pH 6.0) in a pressure cooker. After blocking endogenous peroxidase (3% H_2_O_2_/MeOH for 20 min), all slides were blocked in 10% normal serum/PBST for at least 1 h at RT followed by incubation with primary antibody: rabbit anti-Ki67 at 1:800 (ab15580, Abcam, Cambridge, UK), or rabbit anti-cleaved caspase-3 (9661S @1:200, Cell Signaling Technology) overnight in humid chambers at 4 °C. Slides were developed as described in [39] and imaged with a Keyence BZ-X700 microscope (Keyence, Itasca, IL, USA). Quantification of Ki67 was performed as in [39] and cleaved caspase-3 quantitated as the area of positive cells in 6 representative fields per tissue section (@600× magnification) by a blinded observer. Lung metastasis was evaluated following IHC with an anti-mitochondrial antibody (ab92824, Abcam, Cambridge, UK, 1:1000) and the metastatic burden was quantified by a percent positive area algorithm as described in [51].

### 2.8. Lapatinib IC_50_ Testing and Isobologram Assays to Determine a Drug Combination Index (CI)

BT474, SKBR3 or AU565 (lapatinib-sensitive) and JIMT (lapatinib-resistant) cells were seeded overnight into 96-well plates at a density of either 7500 cells/well (BT474), 5000 cells/well (SKBR3), 15,000 cells/well (AU565) or 8000 cells/well (JIMT) and treated the next day with fresh media containing increasing concentrations of lapatinib alone or, for isobole testing, lapatinib was added in combination with sabizabulin or paclitaxel and cells treated for up to 96 h. The dose range of lapatinib was as follows per cell line: 1 nM to 600 nM for SKBR3 and BT474; 12.5 nM to 10 µM for AU565; 1 nM to 20 µM for JIMT. At the study endpoint, growth inhibition was measured by the MTS assay; drug response was first normalized to untreated vehicle controls and/or to initial seeding density, plotted on a log scale, and then plotted using non-linear regression best fit analysis in GraphPad Prism 9.0. For drug combination studies, the mean IC_30_, IC_50_ and IC_70_ values ± SEM for paclitaxel and sabizabulin were first derived from at least 3 biological replicates. Isobole testing was conducted using the IC_30_ value of sabizabulin or paclitaxel, which was held constant in the presence of increasing concentrations of lapatinib. The CI index was calculated per methods in [52]. Values < 1.0 indicate synergistic drug action, while values of approximating 1.0 are indicative of an additive effect, whereas values > 1.0 suggest antagonism.

### 2.9. Scratch Assay in Response to Drug Combinations

JIMT cells were plated at a density of 38,000 cells/well into a 96-well ImageLock plate (Essen Biosciences) to achieve confluence within 24 h. The following day, the WoundMaker tool was used to scratch the cell monolayer. The cells were washed twice with growth media and then fresh growth media (vehicle) or growth media containing drug were added to the cells in the presence of Cytotox Green (4663, Essen Bioscience) reagent to measure cell viability throughout the wound healing process. Drug doses were based on isobole testing and were selected to be minimally toxic for the duration of the assay time. The plate was imaged every four hours to calculate the wound width. The wound size was normalized to the initial wound width (t = 0 h) and then normalized relative to the untreated, vehicle control. 

### 2.10. Statistical Analysis

Unless otherwise stated, all raw data were entered into Prism 9.0 and analyzed using one-way or two-way ANOVA followed by multiple pairwise comparison tests (*t*-tests). Unless otherwise indicated, on all graphs, the mean ± SEM is shown. Significance was determined at a 95% confidence threshold, and *p*-values indicated by asterisks defined as: * *p* < 0.05, ** *p* < 0.01, *** *p* < 0.001, **** *p* < 0.0001.

## 3. Results

### 3.1. Anti-Proliferative Effect of Sabizabulin on HER2+ Cells In Vitro

**Figure 1:**
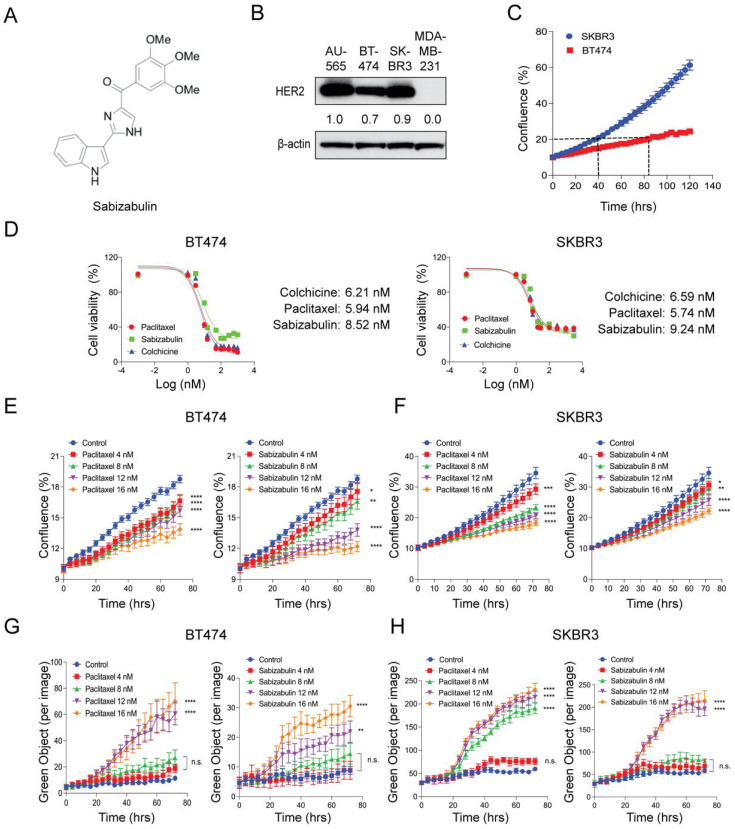
Effect of sabizabulin, colchicine, or paclitaxel in BT474 and SKBR3 HER2+ cell lines. (**A**) Chemical structure of sabizabulin. (**B**) Levels of HER2 in breast cancer cell lines were determined by western blotting; β-actin serves as a loading control. Signal intensity was evaluated by ImageJ densitometry with the AU565 sample set to 1.0. (**C**) Proliferation assay over time (120 h) using BT474 and SKBR3 cells to determine doubling times by confluence (%) using the IncuCyte S3 instrument (5000 cells/96-well; n = 4 wells/cell line). (**D**) The growth inhibition effect of sabizabulin relative to colchicine or paclitaxel was determined by MTS assay after 72 h or 96 h (SKBR3 or BT474, respectively) over a range of concentrations (0.3 nM to 3.0 µM, on a log (nM) scale) and expressed as cell viability (%)representative response curves of three independent biological replicates are shown. (**E**,**F**) Growth inhibition assays for BT474 cells (**E**) or SKBR3 cells (**F**) treated with paclitaxel or sabizabulin at increasing concentrations as measured by confluence (%) over time (hrs). (**G**,**H**) Evaluation of time- and concentration-dependent cytotoxicity for BT474 (**G**) and SKBR3 (**H**) cells as enumerated by Cytotox Green reagent counts (Green Object per image) over time (hrs).All *p*-values are shown as pairwise comparisons to the vehicle (control) group; * *p* < 0.05; ** *p* < 0.01, *** *p* < 0.001, **** *p* < 0.0001; n.s., not significant.

We have previously shown that sabizabulin (Figure 1A) has nanomolar potency in TNBC, with IC_50_ values between 8–9 nM [39]. To compare HER2 levels in cell line models used herein, western blotting was performed. AU565 cells served as a positive control and MDA-MB-231 TNBC cells as a negative control. All models that were selected for evaluation of sabizabulin efficacy express HER2 (Figure 1B). To optimize downstream assays for evaluating growth inhibition, we first characterized each cell line’s doubling time, which was 84 h for BT474 and 40 h for SKBR3 cells, respectively (Figure 1C). In both BT474 and SKBR3 cells, growth inhibition by sabizabulin was observed in the low nanomolar range (Figure 1D and Table 1), with a similar potency observed for both HER2^+^ cell lines.

### 3.2. Sabizabulin Induces Concentration-Dependent Growth Inhibition with Minimal Cytotoxicity

BT474 and SKBR3 cells were exposed to sabizabulin or paclitaxel at increasing concentrations and imaged over time for changes in confluence by phase contrast (Figure 1E,F) while in the presence of Cytotox Green to simultaneously enumerate dead cells (Figure 1G,H). By 72 h, sabizabulin and paclitaxel had significantly inhibited the growth of BT474 and SKBR3 cells in a concentration-dependent manner (Figure 1E,F). Growth inhibition was similar at equivalent concentrations of paclitaxel and sabizabulin for SKBR3 cells (Figure 1F). In contrast, for BT474 cells, sabizabulin was less effective to inhibit growth relative to the same concentrations of paclitaxel. Drug-induced cytotoxicity was significant in BT474 cells at 12 nM or 16 nM (Figure 1G). In contrast, in SKBR3 cells, paclitaxel was cytotoxic beginning at 8 nM, whereas sabizabulin was cytotoxic beginning at 12 nM (Figure 1H). 

### 3.3. Sabizabulin Inhibits Colony Formation in HER2+ Cells

**Figure 2:**
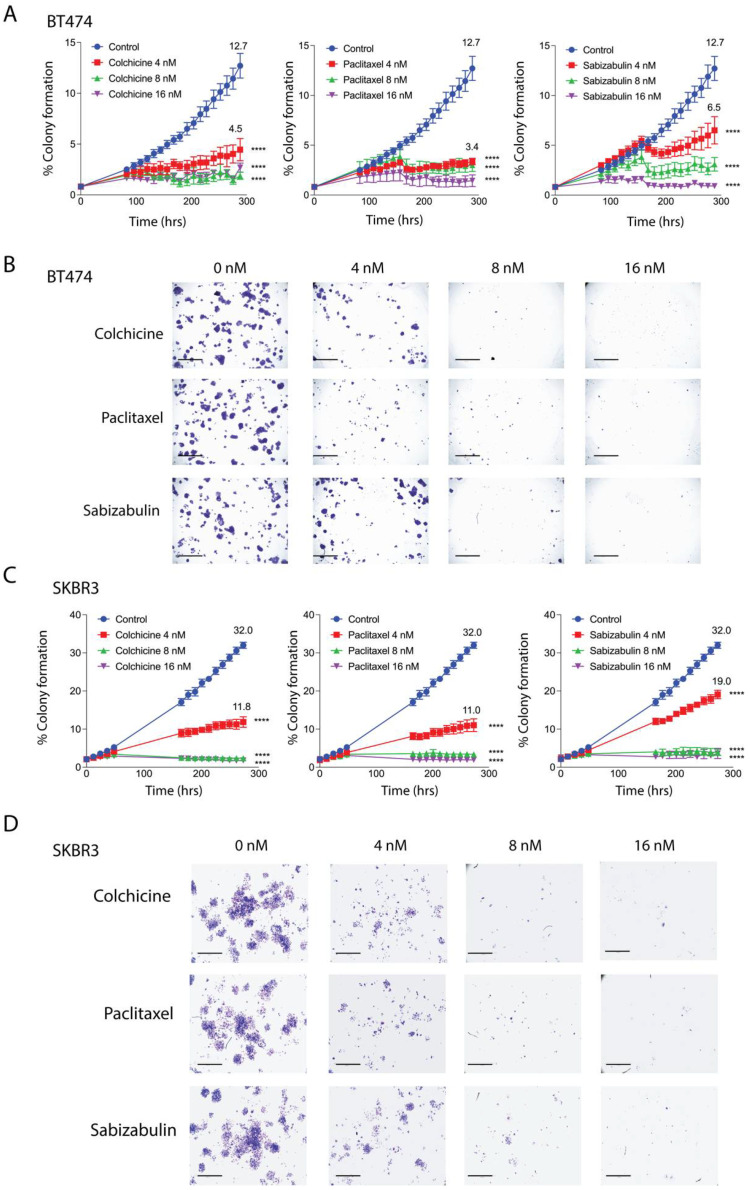
Effect of colchicine, paclitaxel and sabizabulin on colony formation in HER2+ breast cancer cell lines. The growth of colonies was measured for 12 days (288 hrs) in the presence of colchicine, paclitaxel or (4, 8, or 16 nM). (**A**,**B**) The area occupied by colonies over time was converted to a percentage (%) to enumerate colony formation. The mean percent colony formation is indicated at the study endpoint for control and 4 nM treated cells (**A**,**C**). All data represent the grand mean of two independent biological experiments, each assay was performed with n = 8 technical replicates/time point/cell line. Two-way ANOVA was performed followed by Dunnett’s multiple comparisons testing. All *p*-values are shown compared to the vehicle control group at the experimental endpoint; **** *p* < 0.0001. Representative pictures of stained colonies for BT474 (**C**) and SKBR3 (**D**) cells at assay endpoint are shown; scale bars, 50 μM.

BT474 and SKBR3 cells were treated with increasing concentrations of colchicine, paclitaxel, or sabizabulin in a colony forming assay. The phase confluence of vehicle-treated cells at the assay endpoint was set as the maximum colony formation efficiency (100%) and used to calculate the percentage of suppression in drug-treated cells. At a 4 nM concentration, sabizabulin was less effective than paclitaxel in BT474 and SKBR3 cells, whereas at 8 nM and 16 nM concentrations, sabizabulin inhibited clonogenicity relative to vehicle control with similar efficacy to paclitaxel (Figure 2A–D). Overall, sabizabulin significantly inhibited HER2+ breast cancer clonogenicity with higher potency than previously observed for TNBC cell lines [39].

### 3.4. Sabizabulin Induces Apoptosis in HER2^+^ Cells

**Figure 3:**
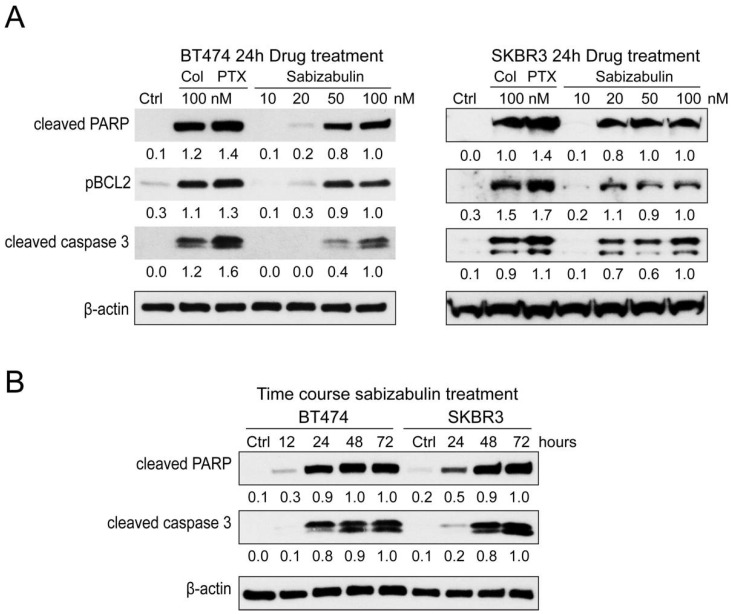
Sabizabulin induces apoptosis in BT474 and SKBR3 cells in a concentration-dependent manner. (**A**) A panel of apoptotic markers was profiled by western blotting after 24 h of treatment with colchicine or paclitaxel (100 nM) or sabizabulin (10, 20, 50, or 100 nM) in BT474 and SKBR3 cells. Signal from cleaved-PARP (anti-cleaved-PARP, CST 5625, 1:1000), phosphorylated BCL2 (pBCL2; anti-pBCL2, 2827 CST 1:1000) and cleaved caspase-3 (CST 9661, 1:500) was first normalized to β-actin (CST 3700, 1:20,000) by ImageJ densitometry, with the 100 nM sabizabulin-treated lane set to 1.0. (**B**) Cleaved-PARP and cleaved-caspase-3 levels in both HER2+ cell lines were determined by western blotting after treatment with 100 nM of sabizabulin for 12, 24, 48, and 72 h after normalization to β-actin. Data are representative of three independent biological replicate assays.

Pro-apoptotic markers were evaluated in BT474 and SKBR3 cells after exposure to colchicine (100 nM), paclitaxel (100 nM), or sabizabulin (10, 20, 50, 100 nM) for 24 h. Sabizabulin induced concentration-dependent upregulation of cleaved PARP, phosphorylated BCL2 (which induces apoptosis), and cleaved caspase-3 (Figure 3A). Induction of apoptosis by 100 nM of sabizabulin was comparable to 100 nM of colchicine, but weaker than observed for paclitaxel. Increasing the duration of sabizabulin exposure increased the induction of cleaved PARP and cleaved caspase-3 proteins in both cell lines (Figure 3B).

### 3.5. Sabizabulin Inhibits BT474 Primary Tumor Growth 

**Figure 4:**
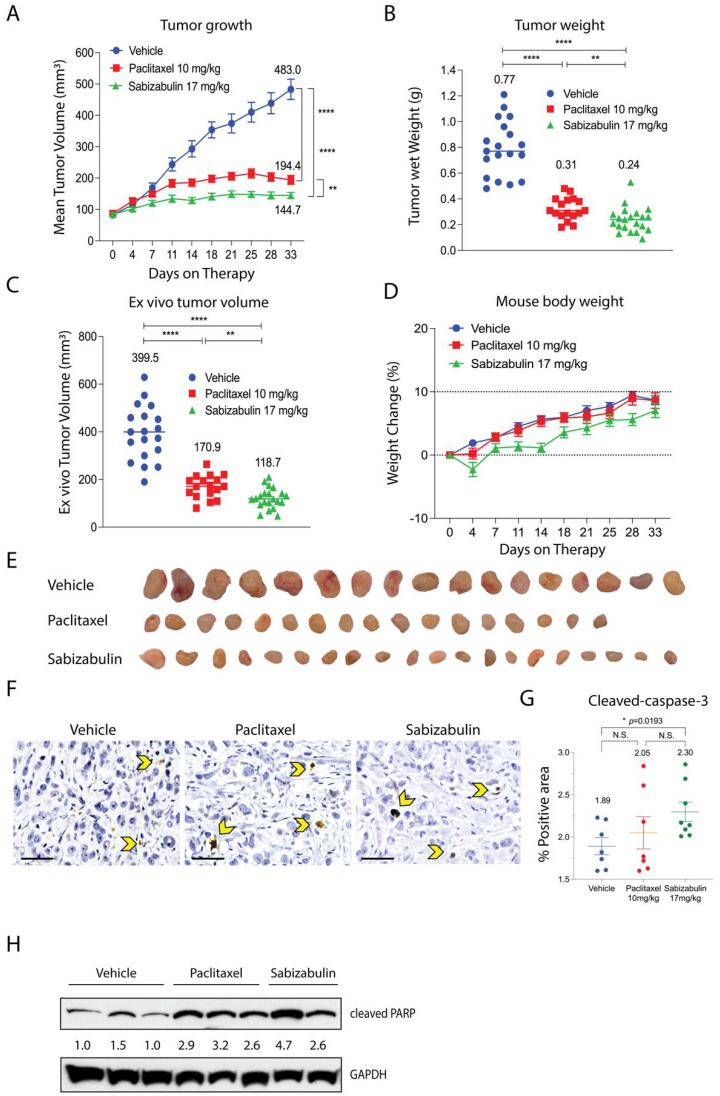
Sabizabulin inhibits tumor growth and induces apoptosis in orthotopic BT474 xenografts. (**A**) Mean tumor volume (mm^3^) was measured over the course of treatments (days on therapy): vehicle (IP, 3×/week, n = 19 total tumors), paclitaxel (10 mg/kg, IP, 3×/week, n = 17 total tumors), or sabizabulin (17 mg/kg, PO, 3×/week, n = 21 total tumors). Mean tumor wet weight (**B**) and mean ex vivo measured tumor volume (**C**) are shown at experiment endpoint (33 days). (**A**–**C**) The mean values are also indicated above each treatment group. All *p*-values were calculated as pairwise comparisons relative to the vehicle control group at 33 days; ** *p* < 0.01; **** *p* < 0.001. (**D**) Mean percent (%) change in body weight over time for vehicle (n = 11 mice), paclitaxel (n = 11 mice), or sabizabulin (n = 12 mice); dashed lines indicate initial body weight (y = 0) with a −10% to +10% variance over time (days on therapy). (**E**) Images of resected tumors. (**F**) Representative images of slides stained with cleaved caspase-3 (600× magnification; scale bar, 50 μM); yellow arrowheads indicate examples of positive cells. (**G**) Quantification of cleaved caspase-3; *p*-values calculated by *t*-tests, with Welch’s correction. N.S., not significant. (**H**) Cleaved PARP signal bywestern blotting of whole tumor extracts with signal normalized to GAPDH (CST 3683) and analyzed by densitometry, with the vehicle-treated signal set to 1.0.

The anti-tumor efficacy of sabizabulin relative to paclitaxel was first assessed in the BT474 orthotopic xenograft model. Sabizabulin (17 mg/kg, PO) significantly inhibited tumor growth relative to vehicle by day 11 of therapy and relative to paclitaxel (10 mg/kg, IP) on day 14 of therapy, with inhibition maintained until the study endpoint (Figure 4A). Sabizabulin significantly reduced end-stage tumor volume and tumor wet weight relative to vehicle-treated animals and was more effective than paclitaxel therapy (Figure 4B,C, Supplemental Figure S1). Minimal toxicity from dosing regimens was evidenced by the increase in animal body weight throughout the study across cohorts (Figure 4D). Images of excised tumors are shown in Figure 4E.

To confirm that sabizabulin induces tumor apoptosis in vivo, sections were immunostained with cleaved caspase-3 antibodies. Significantly more cleaved caspase-3 staining was detected with sabizabulin treatment relative to vehicle treatment. In contrast, there was no significant difference observed between vehicle-treated and paclitaxel-treated tumors, or between paclitaxel- and sabizabulin-treated tumors (Figure 4F,G). Cleaved PARP in tumors was compared by western blotting (Figure 4H). Sabizabulin- and paclitaxel-treated tumors show increased cleaved PARP signal relative to the vehicle control, as expected. 

### 3.6. Sabizabulin Suppresses Tumor Growth and Inhibits Lung Metastases in the HCI-12 HER2+ PDX Model

In general, PDX models replicate clinical metastatic patterns in mice with high genetic similarity to the original patient material, including models derived from the HCI pre-clinical resource [50,53,54]. HCI-12 was selected for evaluation since, unlike BT474 cells, it is ER-/PR- and HER2+ and since it robustly metastasizes to the lungs [50]. 

**Figure 5:**
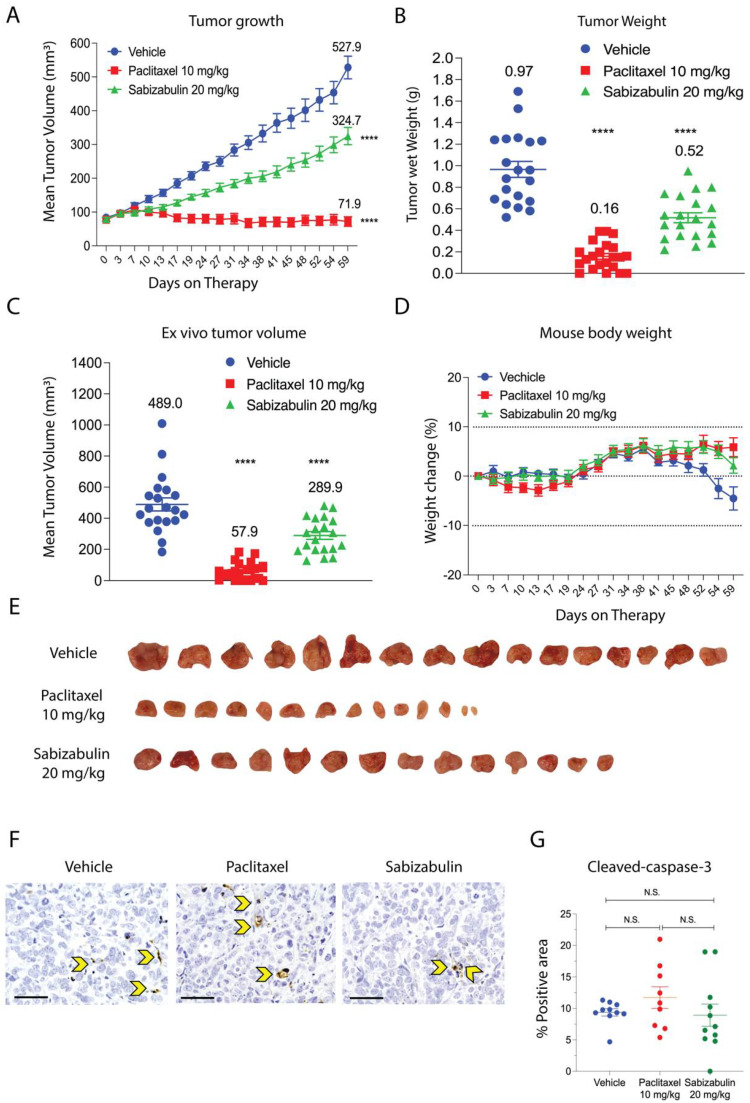
Sabizabulin inhibits primary tumor growth in the HCI-12 PDX model. (**A**) The mean increase in tumor volume (mm^3^) over time on treatment (days on therapy, **A**) and tumor wet weight (grams, g) (**B**) at the study endpoint of59 days after treatment with vehicle (3×/week, IP, n = 21 total tumors), paclitaxel (10 mg/kg; 3×/week, IP, n = 22 total tumors), or sabizabulin (20 mg/kg, 3×/week, PO, n = 18 total tumors). (**C**) The mean ex vivo tumor volume and the percentage increase in body weight for vehicle (n = 11 mice), paclitaxel (n = 11 mice), or sabizabulin (n = 12 mice) relative to body weight at the start of therapy (**D**); the dashed lines indicate initial body weight (y = 0) with a −10% to +10 % variance over time (days on therapy)(**A**–**C**) All pairwise comparisons were made to the vehicle control; **** *p* < 0.001. (**E**) Representative images of resected tumors. (**F**) Representative images of IHC slides stained with cleaved caspase-3 (600× magnification; scale bar 50 μM); yellow arrowheads indicate example apoptotic cells. (**G**) Scatter plot of % positive area for cleaved-caspase-3. N.S. = not significant.

Although HCI-12 PDX is derived from a patient treated with taxanes, its response to paclitaxel in NSG mice was uncharacterized. In contrast to in vitro studies using conventional HER2+ cell lines, in the HCI-12 PDX, paclitaxel was significantly more effective than sabizabulin to repress tumor growth over time (Figure 5A) and at study endpoint (Figure 5B,C), suggesting that HCI-12 PDX model is not paclitaxel-refractory. Whereas paclitaxel completely inhibited tumor growth, sabizabulin was less effective, resulting in a 39% decrease in tumor volume relative to vehicle treatment (Figure 5A). Notably, there was no significant body weight loss at the selected dosing regimens for either drug, suggesting minimal dosing-related toxicity (Figure 5D). Representative tumor images from vehicle, paclitaxel, and sabizabulin treatment groups are shown in Figure 5E. Evaluation of apoptosis by cleaved caspase-3 immumohistochemsitry revealed no significant differences relative to vehicle-treated tumors for paclitaxel or for sabizabulin, or between paclitaxel and sabizabulin (Figure 5F,G). 

**Figure 6:**
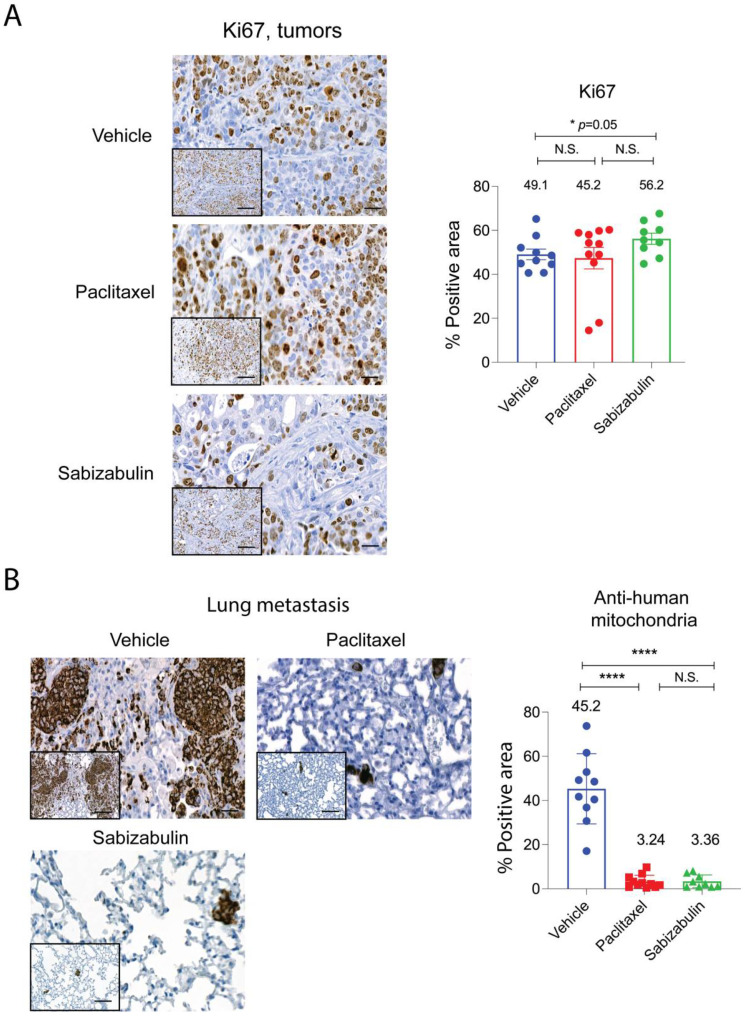
Sabizabulin treatment suppresses lung metastasis as effectively as paclitaxel. Representative images and quantification of signal for Ki67 immunostained tumors (**A**) and lungs stained with an anti-human specific mitochondrial marker to detect metastases (**B**). Images were captured at either low power (200×, inserts; scale bar 50 μM) or higher power magnification (400×; scale bar 20 μM). Areas positive for Ki67 or anti-human mitochondrial staining were quantified after scanning whole tumors (vehicle, n = 10 tumors; paclitaxel, n = 11 tumors; sabizabulin, n = 8 tumors) or whole lungs from each animal (n = 10 vehicle, n = 9 paclitaxel and n = 9 sabizabulin), and the staining intensity analyzed by pixel counts using built-in densitometry software algorithms optimized for nuclear (such as Ki67) localization or cytoplasm localization (such as anti-mitochondria) (3D Histech, Ltd. software, Budapest, Hungary). The percent (%) positive area reflects the number of positive tumor epithelial cells (Ki67) or the % area of metastatic burden (detected by anti-human mitochondria) for whole lungs. All *p*-values are shown relative to the vehicle control group, the mean is indicated above each group; **** *p*< 0.0001; N.S. is not significant.

Since apoptosis was not strongly induced in the HCI-12 PDX model in response to either treatment, we next compared cell proliferation by immunostaining slides for Ki67. Whole stained tumors, after excluding necrotic regions, were subjected to digital scanning and densitometry-based quantification for the Ki67+ tumor area (Figure 6A). Likely because tumors from the vehicle group were highly necrotic, typical of the HCI-12 model, and only healthy tissue was enumerated, the percentage of the positive area for Ki67 was equal to or higher than vehicle group for the treated tumors.

To assay for sabizabulin anti-metastatic activity, lung sections were stained with anti-human specific mitochondria antibodies. On average, in the vehicle group, ~49% of lung area was occupied by metastasis (Figure 6B), whereas in both paclitaxel and sabizabulin cohorts, metastatic burden was < 3.5% of the area of lungs (Figure 6B). Since over 95% of breast cancer patients die from metastasis, it is important to note that sabizabulin was equally effective at inhibiting lung metastases as paclitaxel, despite being far less effective against primary tumor growth. 

### 3.7. Sabizabulin Synergizes with Lapatinib to Inhibit Tumor Cell Growth

**Figure 7:**
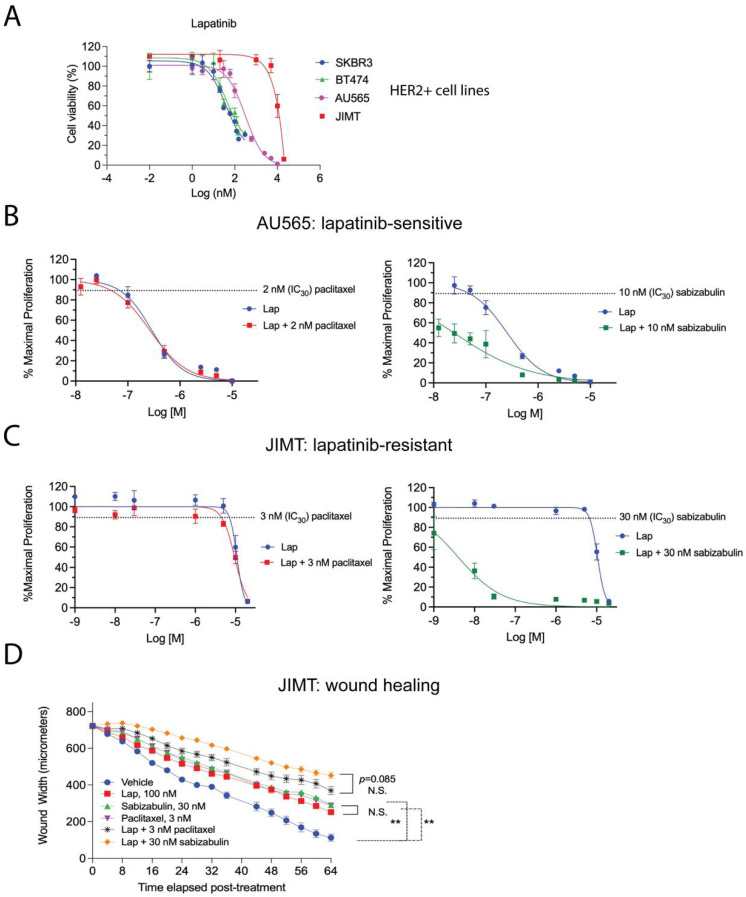
Efficacy of the combination of either paclitaxel or sabizabulin with lapatinib on cell growth inhibition and wound healing. (**A**) Lapatinib sensitivity of BT474, SKBR3, AU565 and JIMT HER2+ cell lines was measured by the MTS assay over a range of doses, data enumerated as a percentage of viable cells, and plotted on log (nM) scale, confirming that JIMT cells are lapatinib-resistant. (**B**,**C**) The MTS assay was used to determine the effect of pairing lapatinib (Lap) with paclitaxel (left panels) or sabizabulin (right panels) in AU565 (**B**) or JIMT (**C**) cells by the isobole method; the effect of the anti-tubulin targeting agent alone at the IC_30_ dose is shown as a dashed line. (**B**,**C**) Data are plotted as the % maximal proliferation on a log scale (M = molar) after normalization to the untreated control as described in the Materials and Methods. Assays in (**A**–**C**) are representative of 3 to 4 biological replicates. (**D**) Wound healing was assayed by measuring wound width over time after drug added using the IncuCyte S3 instrument. JIMT cells were treated with either lapatinib (Lap), sabizabulin or paclitaxel alone, or in combination with 100 nM of lapatinib (n = 8 wells/cell line/treatment). Data are representative of two biological replicate experiments. ** *p* < 0.01; N.S., not significant.

Lapatinib, a reversible EGFR/HER2 tyrosine kinase inhibitor, is paired with capecitabine is a second-line therapy for metastatic HER2+ patients [28]; therefore, we next assayed the lapatinib sensitivity in a panel of HER2+ cell lines. We included AU565 cells and JIMT cells, which, relative to BT474 and SKBR3 cells, are reported to be moderately lapatinib sensitive or lapatinib-resistant, respectively [48]. Growth inhibition was measured by MTS assay in the presence of increasing concentrations of lapatinib, confirming these prior observations (Figure 7A). Next, AU565 (Figure 7B) and JIMT (Figure 7C) cells were used in an isobole assay to determine the combination index (CI) when lapatinib is paired with either paclitaxel or sabizabulin at their respective IC_30_ concentration (paclitaxel = 2 nM for AU565, or 3 nM for JIMT; sabizabulin = 10 nM for AU565, or 30 nM for JIMT) The CI values for each combination tested are reported in Table 2. Whereas the addition of paclitaxel to lapatinib was neither additive or synergistic in either AU565 or JIMT cells, the addition of sabizabulin to lapatinib produced synergistic effects in each cell line, with the lowest CI values observed for lapatinib-resistant JIMT cells. To determine if drug synergy between sabizabulin and lapatinib was also observed during cell migration, a wound healing assay was conducted in JIMT cells with single agents versus combination therapy. The IC_30_ concentrations of either sabizabulin (30 nM) or paclitaxel (3 nM) had equivalent activity to repress wound healing relative to the vehicle control (** *p* < 0.01, either comparison to vehicle). Although there was a trend towards enhanced repression of wound healing with the sabizabulin combination therapy relative to the paclitaxel combination therapy, these data were not statistically significant (*p* = 0.085) (Figure 7D). During the same wound healing experiment shown in Figure 7D, there was minimal cell death over the course of the assay as measured by the incorporation of Cytotox Green dye (Supplementary Figure S2).

## 4. Discussion

HER2+ breast cancer patients are commonly treated with anti-HER2 immunotherapy in conjunction with a tubulin inhibitor [27]. Taxanes are standard of care agents. While initially successful, they are often subject to efflux pump-mediated chemoresistance, they cause neurotoxicity, and they have low aqueous solubility [36,37,38]. CBSIs are exciting potential replacements for taxanes in breast cancer treatment since they are less likely to be susceptible to taxane resistance mechanisms. 

Our group has previously reported the efficacy of a novel CBSI, sabizabulin, in TNBC. Sabizabulin overcomes taxane resistance, is a poor substrate of P-gp mediated drug efflux, is orally bioavailable, and exhibits low levels of toxicity [39,42]. Sabizabulin is highly potent against TNBC both in vitro and in vivo, therefore we determined the efficacy of sabizabulin in HER2+ breast cancer models. The anti-proliferative activity of sabizabulin was determined in two conventional HER2+ breast cancer cell lines, BT474 (ER+/PR+/HER2^+^) and SKBR3 (ER-/PR-/HER2+). The IC_50_ values of sabizabulin for HER2+ cells were in the low nanomolar range, like paclitaxel. Sabizabulin effectively inhibited cell growth without gross cytotoxicity, at sub-IC_50_ concentrations. Clonogenicity assays confirmed the ability of sabizabulin to inhibit colony formation with similar efficacy to paclitaxel. Sabizabulin was also effective to induce apoptosis in vitro, as validated by induction of PARP and caspase-3 cleavage in a dose-dependent and time-dependent manner. Sabizabulin also induced phosphorylation of BCL2, previously shown to correlate with taxane-induced apoptosis [55,56,57,58]. 

In vivo, sabizabulin proved effective in inhibiting HER2+ primary tumor growth, demonstrating higher potency than paclitaxel in BT474 xenografts, but with reduced efficacy relative to paclitaxel in the metastatic HCI-12 HER2+ PDX model. Consistent with these observations, the induction of apoptosis by sabizabulin was higher than paclitaxel in the BT474 model. Although there were no significant differences in apoptosis among the cohorts in the HCI-12 PDX, there was a trend that paclitaxel induced apoptosis relative to the vehicle cohort (*p* = 0.19 for paclitaxel treatment versus *p* = 0.82 for sabizabulin treatment). Since apoptosis was similar in the HCI-12 PDX, despite differences in tumor volumes, we expected that changes in Ki67 would be observed in response to paclitaxel or sabizabulin treatment; however, no significant changes in Ki67 staining of tumor sections were observed among cohorts. It is possible that since those large regions of HCI-12 tumors are necrotic by study endpoint, in contrast to the BT474 xenografts, and since we excluded necrotic regions during immunohistochemistry analyses, we may have underestimated changes in proliferation or apoptosis as assessed by immunohistochemistry in this model.

Since sabizabulin was less effective to repress primary tumor growth than paclitaxel in the HCI-12 PDX, we expected to observe more lung metastatic lesions in the sabizabulin group relative to paclitaxel. However, we observed that sabizabulin was equally effective as paclitaxel to inhibit lung metastasis, despite animals in the sabizabulin cohort bearing larger tumors, which likely seed more cells into the circulation. Since the BT474 model does not robustly metastasize, we could not use it to assay for anti-metastatic potential. Because metastatic lesions that emerge during chemoresistance are a primary driver of metastatic burden that leads to reduced overall survival, it is notable that sabizabulin has equivalent anti-metastatic efficacy as paclitaxel, with the advantage that sabizabulin also overcomes taxane resistance [39]. These results were surprising; since sabizabulin reduced primary tumor volume by only 40% relative to control tumors. 

Preliminary results from our global gene expression and protein target profiling studies of cultured breast cancer cells exposed to paclitaxel versus sabizabulin suggest that sabizabulin has additional molecular targets besides microtubules. Several molecular pathways enriched by sabizabulin appear to be unique to sabizabulin relative to paclitaxel-enriched pathways, i.e., sabizabulin shows likely polypharmacology. These studies, which are still in progress, could also explain why we have observed that sabizabulin is synergistic with lapatinib to inhibit cell growth in HER2+ cells, but paclitaxel is not. In contrast to cell growth, for the wound healing assay, the efficacy of combination therapy with lapatinib on the migratory capacity of JIMT cells was equivalent for paclitaxel and sabizabulin. Therefore, the action of each agent is dependent upon the specific biological phenotype assayed. As potential novel molecular targets of sabizabulin besides microtubules are identified, and the putative direct binding partners are then validated by crystallography, the biological activity of any additional sabizabulin targets may provide an enhanced understanding of the mechanisms of sabizabulin action relative to paclitaxel. 

Based on the potent anti-tumor efficacy in the BT474 model and the anti-metastatic activity in the HCI-12 PDX, further development of sabizabulin for clinical use in HER2+ patients is warranted. In fact, new derivatives have been recently developed based on the sabizabulin scaffold that have activity against a broad panel of cancer types, including taxane-refractory melanoma [59]. Overall, we have shown that sabizabulin is an effective and well-tolerated agent with activity against four HER2+ breast cancer cell lines, BT474, SKBR3, AU565 and JIMT cells, and in two HER2+ xenograft models, and that sabizabulin, but not paclitaxel, sensitizes AU565 and JIMT cells to lapatinib treatment. 

## 5. Conclusions

Pre-clinical studies of sabizabulin to treat prostate cancer and TNBC, and now in HER2+ breast cancer, confirm its safety, its low nanomolar potency, its ability to inhibit primary tumor growth, and lung metastasis, and its ability to induce apoptosis, and evade taxane-resistance [39,42,43,44]. We suggest sabizabulin could be an effective replacement therapy for taxanes, or a well-tolerated second line therapy for patients who acquire taxane resistance. Sabizabulin would also be useful for those patients requiring chemotherapy dose escalation. Side effects of sabizabulin are tolerable; diarrhea and fatigue were observed at a phase II MTD of 63 mg [46]. Sabizabulin could potentially substitute for a taxane in combination with current anti-HER2 therapies, which is an area of future study to determine if there is synergy between sabizabulin (a microtubule destabilizer) with trastuzumab or pertuzumab, as has been observed for paclitaxel (a microtubule stabilizer). Additional future studies of interest include evaluating the efficacy of sabizabulin in acquired taxane-resistant HER2+ breast cancer models, and downstream genetic profiling to discover any conserved mechanisms of taxane resistance that are overcome by sabizabulin in the TNBC and HER2+ breast cancer subtypes.

Sabizabulin is currently under investigation in multiple clinical trials, including a Phase 3 clinical trial for metastatic prostate cancer (NCT04844749) and a Phase 2 trial for metastatic breast cancer (NCT05079360). We will continue to screen for improved sabizabulin analogs that bypass taxane resistance. We will also expand drug development efforts for the conjugation of sabizabulin to tissue-enriched targeting moieties to more effectively and specifically deliver sabizabulin to common sites of breast cancer metastasis in patients that are less frequently observed in pre-clinical rodent models, including the brain, bone, and liver.

## 6. Patents

The following patent covered sabizabulin and some of its related compounds: “Compounds for Treating Cancer”; inventors: Duane D. Miller, Wei Li, Jianjun Chen, James T. Dalton, Chien-Ming Li, Sunjoo Ahn. United State Patent 9,029,408 B2, issued on 12 May 2015.

## Figures and Tables

**Table 1:** Sabizabulin, paclitaxel, colchicine IC_50_ values in HER2+ cells. IC_50_ values ± SEM were determined as described in the Materials and Methods. Results from three independent experiments were averaged to report the grand mean.

	Paclitaxel (nM)	Sabizabulin (nM)	Colchicine (nM)
BT474	7.07 ± 1.14	9.07 ± 0.56	6.14 ± 0.45
SKBR3	6.26 ± 0.52	8.18 ± 1.06	6.90 ± 0.31

**Table 2:** Combinatorial indices (CI) as determined by isobole testing at the IC_30_, IC_50_ and IC_70_ values for paclitaxel or sabizabulin when paired with lapatinib in JIMT and AU565 HER2+ cell lines. Results are representative of three biological replicate isobole assays. Values < 1.0 indicate synergistic drug action, while values of approximating 1.0 are indicative of an additive effect, whereas values > 1.0 suggest antagonism.

	JIMT		AU565	
	Lapatinib + 3 nM Paclitaxel	Lapatinib + 30 nM Sabizabulin	Lapatinib + 2 nM Paclitaxel	Lapatinib + 10 nM Sabizabulin
IC_30_	1.35	0.48	1.49	0.97
IC_50_	1.17	0.22	1.49	0.89
IC_70_	1.14	1.02	1.53	0.88

## Data Availability

The data presented in this study are available on request from the corresponding author.

## Supplementary Materials

The following are available online at https://www.mdpi.com/article/10.3390/cancers14215336/s1. Supplementary Figure S1: Sabizabulin inhibits tumor growth of orthotopic HER2+ BT474 xenografts. Individual tumor measurements for each mouse over time are shown for vehicle (A), paclitaxel (B), and sabizabulin (C) treatment groups during the 33 days on therapy. Supplementary Figure S2: Minimal changes in cell viability are observed during wound healing in JIMT cells. Cytotoxicity was measured by the incorporation of Cytotox Green dye concurrent with the measurements of wound width throughout the same assay shown in Figure 7D. Cytotox Green was normalized at each time point relative to vehicle-treated cells (n = 8 well/treatment/time point), and the mean fold-change ± SEM relative to vehicle reported. No significant changes in cell viability were observed by pairwise *t*-tests with Welch’s correction between any of the groups demarked by the grey bracket. A 2.5 μM dose of paclitaxel (blue line) served as the assay positive control (+control).
