# Supplementary material for: Sabizabulin, a Potent Orally Bioavailable Colchicine Binding Site Agent, Suppresses HER2+ Breast Cancer and Metastasis
Source: Cancers (Basel). 2022 Oct 29;14(21):5336. doi: 10.3390/cancers14215336 (PMC9658816; doi:10.3390/cancers14215336)
Supplement: Supplementary file 1 [file cancers-14-05336-s001.zip › cancers-2006238-supplementary.pdf]

cancers

MDPI

# Supplementary Materials: Sabizabulin, a Potent Orally Bioavailable Colchicine Binding Site Agent, Suppresses HER2+ Breast Cancer and Metastasis

**Raisa I. Krutilina, Kelli L. Hartman, Damilola Oluwalana, Hilaire C. Playa, Deanna N. Parke, Hao Chen, Duane D. Miller, Wei Li and Tiffany N. Seagroves**

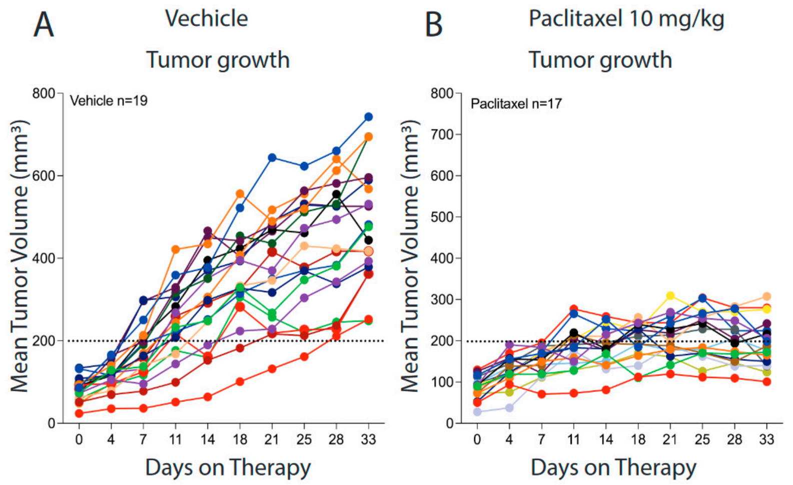


**Figure S1.** Sabizabulin inhibits tumor growth of orthotopic HER2+ BT474 xenografts. Individual tumor measurements for each mouse over time are shown for vehicle (A), paclitaxel (B), and sabizabulin (C) treatment groups during the 33 days on therapy.

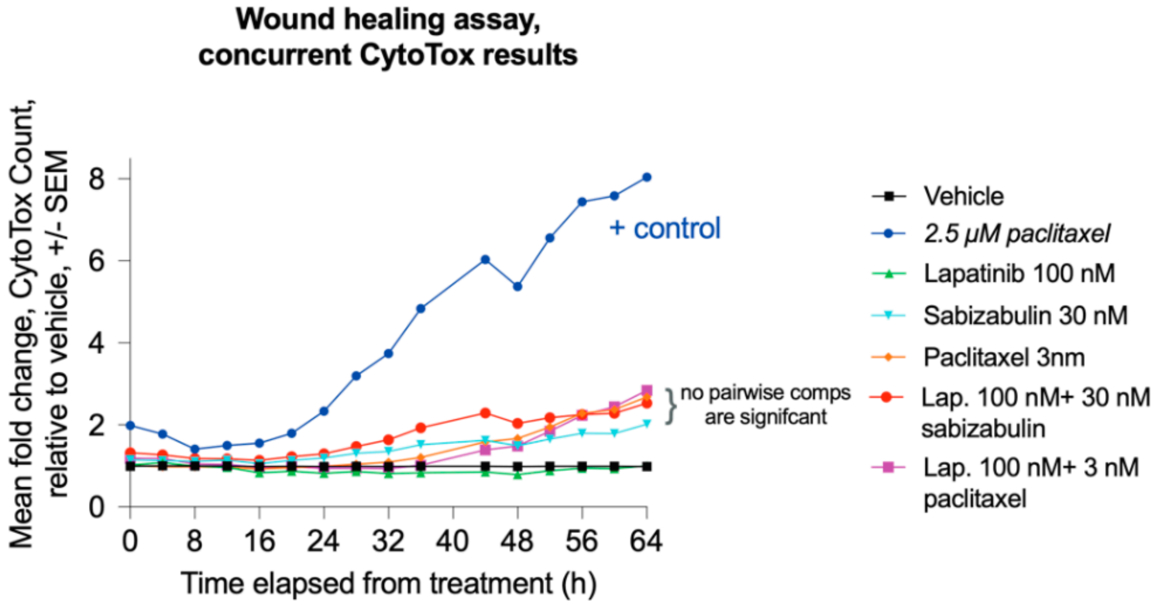


**Figure S2.** Minimal changes in cell viability are observed during wound healing in JIMT cells. Cytotoxicity was measured by the incorporation of Cytotox Green dye concurrent with the measurements of wound width throughout the same assay shown in Figure 7D. Cytotox Green was normalized at each time point relative to vehicle-treated cells (n = 8 well/treatment/time point), and the mean fold-change ± SEM relative to vehicle reported. No significant changes in cell viability were observed by pairwise *t*-tests with Welch's correction between any of the groups demarked by the grey bracket. A 2.5 µM dose of paclitaxel (blue line) served as the assay positive control (+control).
